# Supplementary material for: Causes and consequences of sex-chromosome turnovers in Diptera
Source: Evol Lett. 2026 Mar 12;10(3):253–68. doi: 10.1093/evlett/qrag003 (PMC13231274; doi:10.1093/evlett/qrag003)
Supplement: qrag003_Supplemental_Files [file qrag003_supplemental_files.zip › Supplementary_Datasets.zip!/Supplementary_Datasets/Dataset_5_GC_content_estimation.pdf]

Supplementary Dataset 5: GC content estimatiton of species with a characterized X. P-values were calculated with a Mann-Whitney test

Species	median_x	median_auto	p_value
Acroceridae_Acrocera_orbiculus	0.1921	0.2696	3.59E-12
Anthomyiidae_Pegoplata_infirma	0.3086	0.2954	4.99E-31
Asilidae_Dysmachus_trigonus	0.32615	0.3445	5.20E-15
Asilidae_Eutolmus_rufibarbis	0.33375	0.3441	0.003099658751
Asilidae_Machimus_atricapillus	0.37325	0.3547	2.81E-21
Asilidae_Machimus_rusticus	0.3349	0.3548	2.91E-17
Asilidae_Tolmerus_cingulatus	0.3844	0.35125	6.52E-35
Bibionidae_Bibio_marce	0.30455	0.252	4.49E-47
Bibionidae_Dilophus_febrilis	0.31135	0.2348	5.31E-117
Bombyliidae_Anthrax_anthrax	0.2193	0.2418	9.06E-63
Bombyliidae_Bombylius_discolor	0.2295	0.24275	6.61E-13
Bombyliidae_Bombylius_major	0.2458	0.2524	0.008654773203
Bombyliidae_Villa_cingulata	0.25455	0.2614	1.07E-11
Calliphoridae_Bellardia_pandia	0.32895	0.3009	2.30E-87
Calliphoridae_Calliphora_vomitoria	0.309	0.3074	0.3514951933
Calliphoridae_Protocalliphora_azurea	0.30195	0.2718	1.59E-37
Calliphoridae_Stomorhina_lunata	0.30925	0.2825	1.96E-27
Clusiidae_Clusia_tigrina	0.3801	0.3771	9.80E-18
Coelopidae_Coelopa_pilipes	0.4018	0.433	0.08767792105
Conopidae_Conops_quadrifasciatus	0.3639	0.3536	4.51E-06
Conopidae_Myopa_testacea	0.3666	0.3589	2.32E-39
Conopidae_Sicus_ferrugineus	0.4066	0.4012	1.58E-06
Conopidae_Thecophora_atra	0.3783	0.3694	9.12E-74
Culicidae_Anopheles_albimanus	0.5074	0.4902	0.0001366505714
Culicidae_Anopheles_aquasalis	0.483	0.48635	0.3333493643
Culicidae_Anopheles_arabiensis	0.4644	0.44505	2.58E-16
Culicidae_Anopheles_atroparvus	0.41805	0.4262	0.001217068869
Culicidae_Anopheles_bellator	0.47175	0.4906	9.71E-12
Culicidae_Anopheles_coluzzii	0.4581	0.4456	3.28E-06
Culicidae_Anopheles_coustani	0.4545	0.4449	9.54E-10
Culicidae_Anopheles_cruzii	0.49195	0.4953	0.8052139463
Culicidae_Anopheles_darlingi	0.5072	0.483	4.26E-23
Culicidae_Anopheles_funestus	0.4241	0.4133	1.99E-16
Culicidae_Anopheles_gambiae	0.4546	0.4443	5.72E-07
Culicidae_Anopheles_longipalpis	0.41	0.3926	5.81E-13
Culicidae_Anopheles_maculipalpis	0.4485	0.4293	4.55E-17
Culicidae_Anopheles_marshallii	0.44265	0.4352	0.001662561933
Culicidae_Anopheles_merus	0.4531	0.44485	0.001999130424
Culicidae_Anopheles_moucheti	0.44395	0.4391	0.03380634823
Culicidae_Anopheles_nili	0.47535	0.475	0.5562293313
Culicidae_Anopheles_parensis	0.42	0.39605	9.79E-18
Culicidae_Anopheles_rivulorum*	NA	NA	NA
Culicidae_Anopheles_stephensi	0.4586	0.4487	7.96E-10
Culicidae_Anopheles_vaneedeni	0.4089	0.3496	4.72E-30

Culicidae_Anopheles_ziemani	0.4545	0.4449	9.54E-10
Diopsidae_Teleopsis_dalmanni	0.2994	0.2941	5.84E-12
Dolichopodidae_Dolichopus_griseipennis	0.2546	0.263	3.86E-05
Dolichopodidae_Poecilobothrus_nobilitatuss	0.25295	0.2481	0.2436524861
Drosophilidae_Drosophila_albomicans*	NA	NA	NA
Drosophilidae_Drosophila_ananaese	0.4057	0.4207	1.50E-18
Drosophilidae_Drosophila_athabasca*	NA	NA	NA
Drosophilidae_Drosophila_busckii*	NA	NA	NA
Drosophilidae_Drosophila_elegans*	NA	NA	NA
Drosophilidae_Drosophila_innubila*	NA	NA	NA
Drosophilidae_Drosophila_kepulauana	0.3718	0.38235	2.07E-12
Drosophilidae_Drosophila_Lowei	0.4492	0.4437	1.88E-06
Drosophilidae_Drosophila_mauritiana	0.428	0.42875	0.1805324699
Drosophilidae_Drosophila_melanogaster	0.4209	0.423	0.2961530743
Drosophilidae_Drosophila_miranda	0.453	0.4475	2.24E-07
Drosophilidae_Drosophila_nasuta	0.37185	0.38215	2.50E-14
Drosophilidae_Drosophila_pallidifrons	0.3727	0.3832	4.77E-16
Drosophilidae_Drosophila_sechellia	0.42625	0.42785	0.6111915471
Drosophilidae_Drosophila_subpulchrella*	NA	NA	NA
Drosophilidae_Drosophila_triauraria	0.4099	0.416	1.11E-06
Drosophilidae_Drosophila_virilis	0.41195	0.40465	1.05E-08
Drosophilidae_pseudoobscura_pseudoobscura_d	0.45665	0.4501	1.41E-08
Drosophilidae_sulfurigaster_albostrigata	0.37525	0.3837	3.84E-11
Drosophilidae_sulfurigaster_bilimbata	0.3743	0.3838	1.98E-11
Drosophilidae_sulfurigaster_sulfurigaster	0.3734	0.3837	7.67E-11
Dryomyzidae_Dryomyza_anilis	0.3236	0.3111	3.29E-45
Empididae_Empis_livida	0.24185	0.2359	0.2508786358
Empididae_Rhamphomyia_laevipes	0.2447	0.2512	1.82E-06
Heleomyzidae_Suillia_variegata	0.3303	0.3419	5.65E-08
Hippoboscidae_Ornithomya_chloropus	0.3351	0.3309	4.69E-06
Hippoboscidae_Ornithomya_fringillina	0.3346	0.32405	2.06E-27
Hybotidae_Hybos_culiciformis	0.3209	0.242	2.06E-21
Lauxaniidae_Calliopum_simillimum	0.333	0.3284	8.15E-16
Lauxaniidae_Meiosimyza_decipiens	0.34175	0.3295	2.98E-31
Muscidae_Polietes_domitor	0.5391	0.34	2.70E-64
Pallopteridae_Toxonevra_muliebris	0.33815	0.3257	3.47E-37
Pipunculidae_Nephrocerus_scutellatus	0.37755	0.3803	0.01892960599
Polleniidae_Pollenia_amentaria	0.3214	0.3042	1.35E-52
Polleniidae_Pollenia_angustigena	0.31745	0.3116	1.23E-17
Ptychopteridae_Ptychoptera_albimana	0.2956	0.2401	2.94E-21
Ptychopteridae_Ptychoptera_contaminata	0.2584	0.23095	1.86E-12
Rhagionidae_Rhagio_lineola	0.3291	0.3311	1.03E-06
Rhinophoridae_Melanophora_roralis	0.336	0.3138	2.16E-103
Rhinophoridae_Phyto_melanocephala	0.3158	0.2993	6.24E-61
Sarcophagidae_Sarcophaga_caerulescens	0.33155	0.33235	0.898648907
Sarcophagidae_Sarcophaga_roselle	0.3323	0.3341	0.07244970155

Sarcophagidae_Sarcophaga_subvicina	0.33555	0.3348	0.6126729728
Sarcophagidae_Sarcophaga_variegata	0.33595	0.335	0.8980405593
Sciaridae_Bradysia_coprophila	0.3465	0.3564	4.83E-48
Sciaridae_Pseudolycoriella_hygida	0.3625	0.368	2.17E-39
Sciomyzidae_Coremacera_marginata	0.3386	0.3356	0.07721261524
Sciomyzidae_Pherbina_coryleti	0.3371	0.335	0.07413268669
Sciomyzidae_Tetanocera_ferruginea	0.3219	0.3236	0.2375943027
Stratiomydae_Beris_chalybata	0.3912	0.3654	6.12E-28
Stratiomydae_Beris_morrisii	0.3921	0.3651	5.28E-30
Stratiomydae_Chorisops_tibialis	0.4075	0.3698	3.97E-58
Stratiomydae_Nemotelus_nigrinus	0.386	0.3656	1.54E-32
Stratiomydae_Odontomyia_ornata	0.40375	0.3899	1.33E-71
Syrphidae_Blera_fallax	0.3852	0.39185	0.0007577297792
Syrphidae_Brachypalpus_laphriformis	0.4292	0.4113	4.73E-28
Syrphidae_Cheilosia_impressa	0.3816	0.3826	0.1466019985
Syrphidae_Cheilosia_pagana	0.3782	0.3803	0.2389005662
Syrphidae_Cheilosia_soror	0.3763	0.38055	0.0005970635318
Syrphidae_Cheilosia_urbana	0.3829	0.3768	8.22E-08
Syrphidae_Cheilosia_vulpina	0.3796	0.373	2.96E-05
Syrphidae_Chrysotoxum_bicinctum	0.33355	0.3398	1.33E-06
Syrphidae_Criorhina_berberina	0.3922	0.4134	2.65E-36
Syrphidae_Dasysyrphus_albostriatus	0.3404	0.3325	5.77E-17
Syrphidae_Epistrophe_grossulariae	0.325	0.3175	4.11E-05
Syrphidae_Epistrophella_euchroma	0.3296	0.3262	0.01740559806
Syrphidae_Episyrphus_balteatus	0.3041	0.3096	0.0001056631675
Syrphidae_Eristalinus_sepulchralis	0.4011	0.4146	0.07426590803
Syrphidae_Eristalis_arbustorum	0.4555	0.435	0.001202477465
Syrphidae_Eristalis_pertinax	0.4036	0.4273	0.001320373825
Syrphidae_Eristalis_tenax	0.43205	0.4236	0.0182998631
Syrphidae_Eupeodes_corollae	0.31835	0.3386	2.90E-20
Syrphidae_Eupeodes_latifasciatus	0.3437	0.3332	2.44E-44
Syrphidae_Leucozona_laternaria	0.33985	0.3443	0.04293128877
Syrphidae_Melanostoma_mellinum	0.3681	0.3694	0.02484071511
Syrphidae_Meliscaeva_auricollis	0.3405	0.3259	4.26E-20
Syrphidae_Myathropa_florea	0.41895	0.4135	0.03639071229
Syrphidae_Neoascia_interrupta	0.39395	0.3906	0.002206314847
Syrphidae_Platycheirus_albimanus	0.33985	0.3529	5.66E-37
Syrphidae_Rhingia_campestris	0.379	0.3832	2.02E-05
Syrphidae_Scaeva_pyrastri	0.3204	0.3245	0.01192092716
Syrphidae_Sphaerophoria_taeniata	0.3839	0.3649	6.51E-16
Syrphidae_Syritta_pipiens	0.3889	0.38495	0.8328037843
Syrphidae_Volucella_inanis	0.3657	0.3754	1.95E-45
Syrphidae_Volucella_inflata	0.381	0.389	6.12E-12
Syrphidae_Xanthogramma_pedissequum	0.3438	0.3361	2.75E-21
Syrphidae_Xylota_sylvarum	0.38265	0.385	0.5099847686
Tachinidae_Cistogaster_globosa	0.3179	0.2935	3.32E-79

Tachinidae_Cochliomyia_hominivorax	0.2864	0.2774	1.13E-05
Tachinidae_Epicampocera_succincta	0.3331	0.3285	0.0001868010809
Tachinidae_Gymnosoma_rotundatum	0.3216	0.2995	7.10E-45
Tachinidae_Lypha_dubia	0.3403	0.3048	3.73E-102
Tachinidae_Nowickia_ferox	0.3151	0.311	4.02E-05
Tachinidae_Tachina_fera	0.31745	0.3011	2.11E-31
Tachinidae_Tachina_lurida	0.3209	0.3119	4.26E-22
Tachinidae_Thecocarcelia_acutangulata	0.3398	0.3261	1.21E-25
Tachinidae_Thelaira_solivaga	0.33165	0.2939	1.53E-127
Tephritidae_Anastrepha_ludens	0.3846	0.37015	2.34E-138
Therevidae_Thereva_nobilitata	0.3456	0.3716	5.29E-28
Tipulidae_Nephrotoma_appendiculata	0.3475	0.3311	2.47E-35
Tipulidae_Nephrotoma_flavescens	0.3323	0.328	0.00119453311

*Due to a discrepancy of what is characterized as X in the assembly and what the literature reports as X-linked, or due to entire set of a Muller element assigned as unplaced scaffolds, we did not estimate GC content.
